# Precision in bipolar disorders

**DOI:** 10.1192/j.eurpsy.2023.33

**Published:** 2023-07-19

**Authors:** I. Grande

**Affiliations:** Hospital Clínic, Barcelona, Spain

## Abstract

**Abstract:**

In many fields of heath, precision medicine has been integrated in clinical practise, for instance in oncology. In the case of psychiatry, and in particular bipolar disorder, we are in a prior step for the moment. Different proposal of stratification will be described during the talk for instance the classification of staging. Moreover some proposal of precision psychiatry in bipolar disorder will be put forward.

**Disclosure of Interest:**

None Declared

